# Host-directed treatments for tuberculous meningitis: A multi-platform approach across mouse and human models

**DOI:** 10.21203/rs.3.rs-8694483/v1

**Published:** 2026-03-08

**Authors:** Sanjay Jain, Carlos Ruiz Gonzalez, Medha Singh, Yuderleys Masias Leon, Xueyi Chen, Mona Sarhan, Yazmin Martinez-Martinez, Shruti Patel, Madelynn Shambles, Andres Villabona-Rueda, Kadia Lissit, David Tweedie, Michael Scerba, William Bishai, Dmitri Artemov, Jinchong Xu, Franco D’Alessio, Richard Hafner, Nigel Greig, Maura Manion, Irini Sereti

**Affiliations:** Cincinnati Children’s Hospital Medical Center; Cincinnati Children’s Hospital Medical Center; Cincinnati Children’s Hospital Medical Center; Cincinnati Children’s Hospital Medical Center; Johns Hopkins University School of Medicine; Cincinnati Children’s Hospital Medical Center; Johns Hopkins University School of Medicine; Johns Hopkins University School of Medicine; Johns Hopkins University School of Medicine; Miller School of Medicine, University of Miami; Johns Hopkins University School of Medicine; National Institutes of Health; National Institutes of Health; Johns Hopkins University, School of Medicine, Department of Medicine, Center for Tuberculosis Research, Baltimore; Johns Hopkins University School of Medicine; Johns Hopkins Medicine; Miller School of Medicine, University of Miami; National Institutes of Health; NIA; National Institute of Allergy and Infectious Diseases; NIAID

## Abstract

Tuberculous meningitis (TB meningitis) is a major cause of death and neurological deficit despite recommended antibiotic and corticosteroid treatments, primarily due to dysregulated neuroinflammation. Here, we investigate a diverse panel of 12 immunomodulatory drugs as host-directed treatments (HDTs) for TB meningitis utilizing a cross-species framework comprising studies in a mouse model of TB meningitis with clinical endpoints, and parallel mechanistic studies in a newly developed immune-vascularized human brain organoid model of TB meningitis and peripheral blood mononuclear cells (PBMCs) from patients with TB meningitis. We identify new HDTs that outperform the current standard of care by reducing mortality and neurological deficits in mice via suppression of neuroinflammation. Importantly, these HDTs significantly reduce microglial activation in *Mycobacterium tuberculosis*-infected human brain organoids and attenuate proinflammatory cytokines, particularly IFNγ within CD4 + T-cells in patient-derived PBMCs. These findings highlight the potential of targeted HDTs to improve outcomes in TB meningitis and warrant clinical investigation.

## INTRODUCTION

Despite recent reductions in tuberculosis (TB) incidence rates, TB has once again resurged as a leading cause of mortality from a single infectious agent, surpassing coronavirus disease (COVID-19)^1^. Tuberculous meningitis (TB meningitis) is the most severe form of TB^2^. Despite appropriate antibiotic treatment, at least one of four patients with TB meningitis dies, with mortality of 67% in Asia and up to 80% in Sub-Saharan Africa^2,3^. Furthermore, neurological sequelae are reported in 29–51% of the surviving patients^4,5^.

Disease severity and outcomes for TB meningitis are largely driven by a dysregulated immune response within the central nervous system (CNS)^6–8^. While these immune mechanisms are essential for mycobacterial control, they lead to exudative meningitis, endovasculitis, infarction, hydrocephalus, and irreversible neuronal damage. Efforts to improve patient outcomes by controlling inflammation in TB meningitis date back to 1952, with the first description of corticosteroids as host-directed treatment (HDT)^9^. Since then, numerous clinical trials have investigated this approach demonstrating that adjunctive corticosteroids result in a 25% reduction in mortality in patients with TB meningitis, although no significant benefit has been observed in reducing neurological deficits^10^. Additionally, the benefits of corticosteroids for TB meningitis in patients living with HIV (PLWH) remains unclear^11^. Importantly, and contrary to results from phase II clinical trials^12–15^, two recent randomized clinical trials highlight the challenges of corticosteroids for TB meningitis. Meya *et al.* highlight rifampin-induced hepatic clearance of corticosteroids altering their efficacy in patients with TB meningitis^16^, and Donovan *et al*. demonstrate no difference in mortality or neurological outcomes in genotype-stratified [leukotriene A4 hydrolase (LTA4H), leukotriene pathway] TB meningitis patients expected to benefit differentially from adjunctive corticosteroids^17^. Together, these findings underscore a critical unmet therapeutic need for developing new HDTs for TB meningitis.

At least two other immunomodulatory drugs are being evaluated in clinical studies for TB meningitis. These include aspirin that modulates cyclooxygenase (COX) activity^18^ and infliximab, a monoclonal antibody against TNFα^19^. The immunomodulatory imide drug (IMiD) thalidomide, which has multiple modes of action including TNFα inhibition, has also shown promise in observational studies in children^20^, but is not widely used^21^.

**Figure 1:**
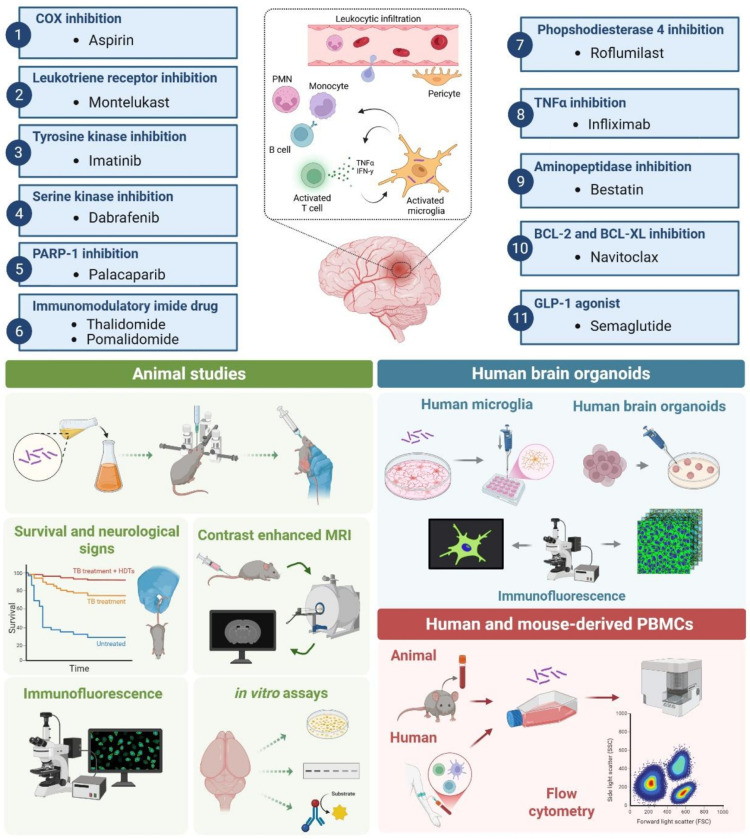
Model systems. We evaluated a panel of 12 immunomodulatory drugs, representing 11 different immunomodulatory drug classes, which are U.S. FDA-approved or in clinical trials for other indications, targeting diverse host-immune pathways, as potential HDTs for TB meningitis. Multiple model systems were utilized, including an established mouse model of TB meningitis using clinical endpoints [mortality, neurological deficits, dynamic contrast-enhanced magnetic resonance imaging (MRI)]; a newly developed 3D immune-vascularized human brain organoid model of TB meningitis, and human-derived peripheral blood mononuclear cells (PBMCs) from patients with TB meningitis. COX, cyclooxygenase; PARP-1, Poly(ADP-ribose) polymerase 1; BCL-2, B-cell lymphoma 2; BCL-XL, B-cell lymphoma-extra-large); GLP-1, Glucagon-like peptide-1. Created in BioRender. Masias, Y. (2026) https://biorender.com/mpr1v9l

In this study, we evaluated a diverse panel of 12 immunomodulatory drugs, representing 11 different immunomodulatory drug classes, which are either U.S. FDA-approved or in clinical trials for other indications, as potential HDTs for TB meningitis (Fig. 1). We employed an integrated cross-species framework that combined longitudinal preclinical studies in an established mouse model of TB meningitis utilizing clinical endpoints [mortality, neurological deficits, dynamic contrast-enhanced magnetic resonance imaging (MRI)] with parallel mechanistic studies in a newly developed 3D immune-vascularized human brain organoid model of TB meningitis and peripheral blood mononuclear cells (PBMCs) from patients with TB meningitis. Studies in the human brain organoid model and PBMCs from patients with TB meningitis allowed us to respectively assess the effect of promising immunomodulatory drugs on resident human brain immune cells (in particular microglia) and on peripheral immune cells, which infiltrate the CNS in active TB meningitis and play a critical role in the pathogenesis of TB meningitis^22–24^. We identified several new immunomodulatory drug classes that reduce both mortality and neurological deficits in mice compared to the current standard of care, with associated decrease in neuroinflammation. Importantly, when added to the standard, first-line TB treatment (isoniazid + rifampin + pyrazinamide), these HDTs reduced microglial activation in *Mycobacterium tuberculosis*-infected human brain organoids and attenuated critical proinflammatory cytokines, particularly IFNγ within CD4 + T-cells in patient-derived PBMCs. Our data strongly suggest the potential role for these immunomodulatory drugs as HDTs for TB meningitis rendering support for evaluation in clinical trials for TB meningitis.

## RESULTS

We used an established mouse model of TB meningitis^25–27^, utilizing intraventricular injection with *M. tuberculosis*. Two weeks after infection, these mice demonstrated hallmark features of TB meningitis, including parenchymal lesions, meningeal infiltration and activated microglial and astrocyte morphology (**Fig. S1**).

### Mortality and neurological deficits in the mouse model of TB meningitis

**Figure 2:**
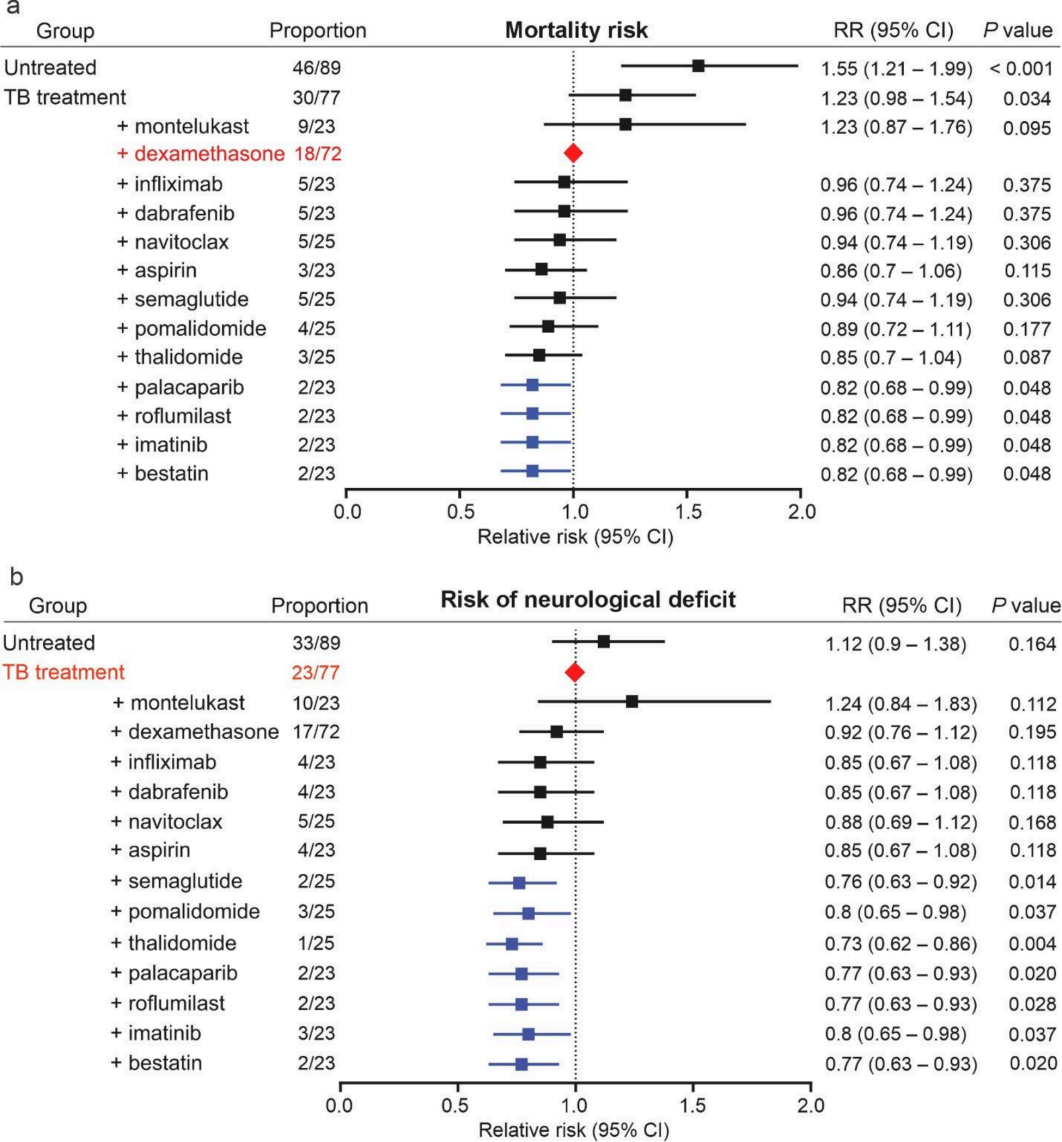
Mortality and neurological deficits in the mouse model of TB meningitis. Two weeks after an intraventricular infection with *M. tuberculosis*, mice were randomly assigned to receive TB treatment (isoniazid) with or without adjunctive host-directed treatment (HDT) for two weeks at human equipotent and clinically relevant dosing for each drug. Mortality and the development of neurological deficits were monitored for 3 months after treatment discontinuation, with the investigators blinded to treatment assignments. (**a**) Relative risk (RR) of mortality and 95% confidence intervals (CI) using TB treatment with adjunctive dexamethasone as the reference (control) arm, based on clinical evidence that adjunctive dexamethasone reduces mortality in TB meningitis. (**b**) RR of developing neurological deficits (head tilt or limb paralysis) and 95% confidence intervals (CI) using TB treatment as the reference (control) arm, based on clinical evidence that adjunctive dexamethasone does not reduce the development of neurological deficits in TB meningitis. Animal numbers are indicated for each treatment arm. 95% CI and statistical significance were assessed using the Katz log-rank test and the Chi-square test.

Two weeks after infection, mice were randomly assigned to receive TB treatment (isoniazid) with or without one of the 12 immunomodulatory drugs or dexamethasone for two weeks at human equipotent and clinically relevant dosing for all drugs (**Table S1**). This approach was utilized to rank order the effects of each immunomodulatory drug during the initial phase (2-weeks) of TB treatments, associated with high mortality in patients with TB meningitis, and thus the ideal time for modulating host responses^12,22,28,29^. Each immunomodulatory drug was administered in combination with isoniazid (TB antibiotic highly active during the initial phase of treatment) as is common for the initial evaluation of new HDTs^30,31^. Mortality as well as the development of neurological deficits was monitored for an additional 3 months, with the investigators blinded to the treatment assignments (**Fig. S2-S5**). The relative risk (RR) of mortality or the development of neurological deficits was calculated for each treatment arm. Consistent with its effects in patients with TB meningitis^32^, adjunctive use of dexamethasone in mice reduced mortality (*P* = 0.030; Fig. 2a, S3), but did not reduce neurological deficits (*P* = 0.195; Fig. 2b) when compared to TB treatment alone. Therefore, the dexamethasone arm was used as the reference (control) for mortality and the TB treatment alone arm was used as the reference (control) for neurological deficits.

As expected, no mortality was noted in sham controls (animals injected intraventricularly with PBS) and untreated animals had a significantly higher mortality (*P* < 0.001) compared to the reference (adjunctive dexamethasone treatment) arm. Adjunctive treatment with bestatin, imatinib, roflumilast, or palacaparib led to a significant reduction in mortality compared to the reference (adjunctive dexamethasone treatment) arm (*P* < 0.048). Adjunctive treatment with other HDTs were not different compared to the reference arm (Fig. 2a), although adjunctive treatment with thalidomide, aspirin, pomalidomide, semaglutide, or navitoclax led to a significant reduction in mortality compared to the TB treatment alone arm (*P* < 0.040) (**Fig. S3**).

As expected, no neurological deficits were noted in sham controls and untreated animals had more neurological deficits compared to the TB treatment alone control arm (**Fig. S5**). Adjunctive treatment with bestatin, imatinib, roflumilast, palacaparib, thalidomide, pomalidomide, or semaglutide led to a significant reduction in neurological deficits compared to the reference (TB treatment alone) arm (*P* < 0.037) (Fig. 2b, **Fig. S5**).

Overall, we identified that adjunctive use of bestatin (protease inhibitor), imatinib (tyrosine kinase inhibitor), roflumilast [phosphodiesterase-4 inhibitor (PDE-4)], or palacaparib [Poly(ADP-ribose) polymerase 1, PARP-1 inhibitor] significantly reduced both mortality (compared to the control arm, adjunctive dexamethasone) and neurological deficits (compared to the control arm, TB treatment alone). Adjunctive use of thalidomide/pomalidomide (IMiD) and semaglutide [Glucagon-like peptide-1 (GLP-1) receptor agonist] significantly reduced neurological deficits (compared to the control arm, TB treatment alone) but were no different than dexamethasone in reducing mortality. Of note, neurological deficits observed for thalidomide/pomalidomide (IMiD) arms were amongst the lowest (RR 0.73 and 0.80, respectively). Adjunctive use of aspirin (COX inhibitor) or navitoclax [B-cell lymphoma 2 (BCL-2) and B-cell lymphoma-extra-large (BCL-XL) inhibitor – proapoptotic^33^] led to a significant reduction in mortality compared to the TB treatment alone arm (*P* < 0.040) (**Fig. S3**). Adjunctive use of infliximab (monoclonal antibody against TNFα) did not reduce mortality (compared to the control arm, adjunctive dexamethasone; or TB treatment alone arm), nor did it reduce neurological deficits (compared to the control arm, TB treatment alone). Finally, montelukast (leukotriene receptor inhibitor) paradoxically increased mortality and neurological deficits. Based on these data, we selected seven immunomodulatory drugs, bestatin, imatinib, roflumilast, palacaparib, pomalidomide (more potent than thalidomide^34^), semaglutide, and aspirin for further evaluation. Aspirin was chosen as it reduced mortality compared to the TB treatment alone arm (although not compared to the control, adjunctive dexamethasone arm), and as it is currently being evaluated in clinical trials for TB meningitis^18^.

### Bactericidal activity and target engagement for the Immunomodulatory drugs

Immunomodulatory drugs could directly affect *M. tuberculosis* killing and modulation of host responses could affect (increase or decrease) bacterial killing^35^. To address this, organs (brain, lung and spleen) from a cohort of infected mice were collected two weeks after treatment initiation. The tissues were homogenized and plated to assess bacterial burden as colony forming units (CFU). As expected, TB treatment with isoniazid (antibiotic) alone was associated with a reduction in CFU compared to untreated animals in the brain, lungs, and spleen. However, the addition of none of the 12 immunomodulatory drugs or dexamethasone, significantly altered the organ bacterial burden in the brain, nor increased dissemination to the lungs or spleen (**Fig. S6**).

Some drugs fail to achieve their effects due to inadequate tissue levels. Drug doses and their brain effects were available for aspirin^36^ and montelukast^37^, and were used for the current studies. For other small molecules, Western blots were performed on mouse whole-brain lysates from the treatment cohorts (**Fig. S6**), demonstrating pathway-appropriate modulation: semaglutide downregulated GLP-1 expression, roflumilast, imatinib, navitoclax and bestatin, respectively decreased PDE-4, PDGFRα/β, BCL2, and CD13, while palacaparib increased PARP-1 cleavage, consistent with its role in inducing apoptosis. Thalidomide and pomalidomide reduced cereblon expression. Collectively, these data confirm that these drugs engage their intended molecular targets within the brain during TB meningitis (**Fig. S7**).

### Dynamic contrast-enhanced brain MRI in the mouse model of TB meningitis

Next, we evaluated the effects of the seven promising HDT classes on neuroinflammation using dynamic contrast-enhanced brain MRI in a cohort of *live* animals, complemented by post-mortem studies 2-weeks after treatment initiation. Contrast-enhanced imaging can localize areas of local tissue inflammation due to the extravasation of intravenous contrast from inflamed (and leaky) blood vessels^38^. MRI was performed using custom-built MRI compatible biocontainment beds for imaging live animals under biosafety level-3 (BSL-3) containment (**Fig. S8**). Dynamic contrast enhanced MRI demonstrated contrast enhancement in the periventricular region (site of infection) in infected mice, which persisted throughout the duration of the scan (20 min). No such enhancement was noted in the sham controls (injected with sterile PBS) (**Fig. S9**). The MRI contrast signal was quantified as the area under the curve (AUC) and normalized to background levels. Delta maps were generated by digitally subtracting the pre-contrast from the post-contrast images (**Fig. S10**).

**Figure 3:**
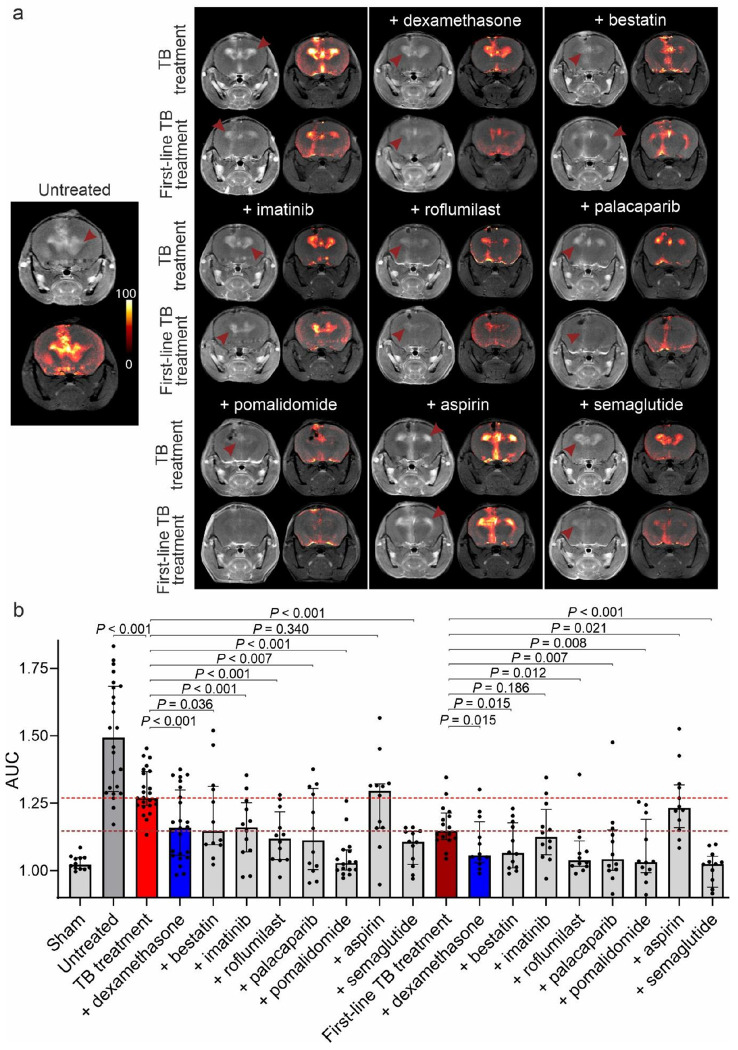
Dynamic gadolinium-enhanced brain MRI in the mouse model of TB meningitis. (**a**) Representative axial T1-weighted MRI images acquired post-contrast injection (left column) with corresponding delta maps (right column) performed at two weeks after treatment initiation in mice showing contrast enhancement in the periventricular region are shown. TB treatment (isoniazid) or first-line TB treatment (isoniazid + rifampin + pyrazinamide) with or without adjunctive HDT were administered at human equipotent and clinically relevant dosing. (**b**) MRI contrast signal was quantified as the area under the curve (AUC) and normalized to background levels. Delta maps were generated by digitally subtracting the pre-contrast image from the post-contrast segmented brain image. Each dot represents a single region of interest (ROI), with 3 brain ROIs drawn for each animal. The dashed line represents the median values for the TB treatment (red line) and the first-line TB treatment (burgundy line) arms. Data are presented as median and interquartile range (IQR). Statistical comparisons were made using the Mann-Whitney U test.

Initial studies utilized TB treatment (isoniazid) with or without one of the seven promising HDTs or dexamethasone. TB treatment alone significantly reduced the contrast enhancement compared to untreated animals (*P* < 0.001), which was further reduced in the dexamethasone arm, compared to TB treatment alone (*P* < 0.001) (**Fig. S11**). Importantly, bestatin, imatinib, roflumilast, palacaparib, pomalidomide, or semaglutide (*P* < 0.040) arms, but not aspirin (*P* = 0.34) or montelukast (signal higher), demonstrated significant reductions in contrast enhancement compared to TB treatment alone (Fig. 3a, c). Similar trends were noted in studies utilizing first-line TB treatment (isoniazid + rifampin + pyrazinamide) with or without adjunctive HDTs. As expected, the first-line TB treatment was more efficacious than isoniazid alone in reducing contrast enhancement. All HDTs arms tested (*P* < 0.020), except for aspirin (signal higher) and imatinib (*P* = 0.186), demonstrated further and significant reductions in contrast enhancement compared to the first-line TB treatment alone (Fig. 3b, c).

### Brain tissue inflammation in the mouse model of TB meningitis

**Figure 4:**
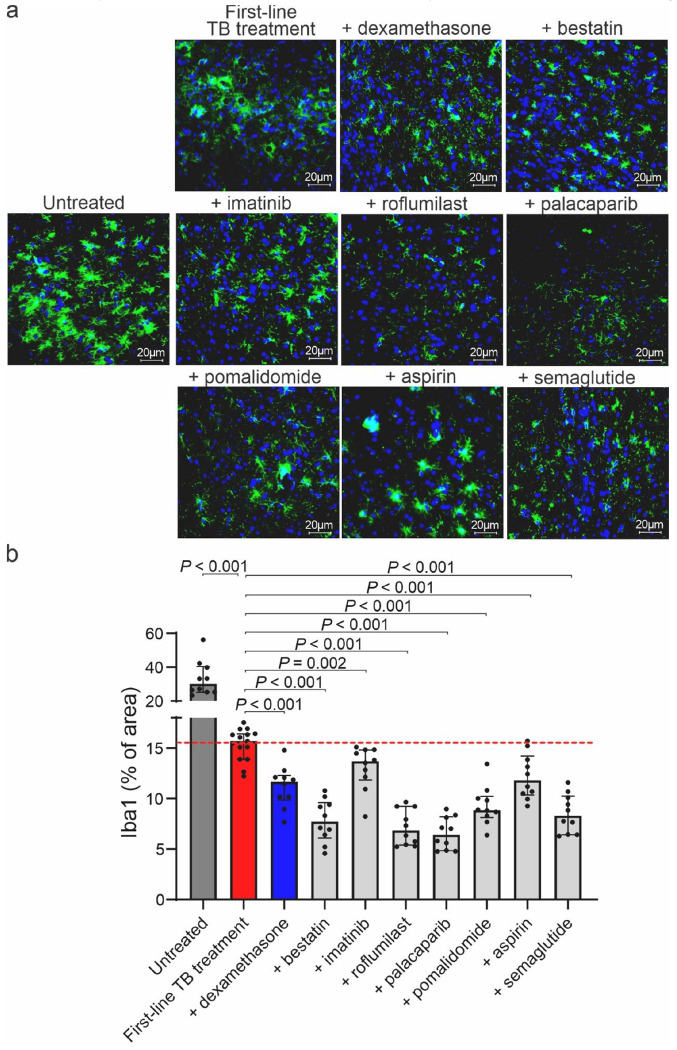
Brain tissue studies in the mouse model of TB meningitis. Brain tissues were collected, fixed, and stained two weeks after treatment initiation in mice receiving first-line TB treatment (isoniazid + rifampin + pyrazinamide), with or without adjunctive HDTs. (**a**) Representative immunofluorescence images are shown. Microglia are labeled with ionized calcium-binding adaptor molecule 1 (Iba1, green), and nuclei are counterstained with 4’,6-diamidino-2-phenylindole (DAPI, blue) (40x magnification, scale bar = 20 μm). (**b**) Quantification of Iba1+ (percentage of area covered by positive staining) for each treatmentarm. Each dot represents a single brain section. The dashed line represents the median value for the first-line TB treatment arm. Data are presented as median and IQR. Statistical comparisons were performed using the Mann-Whitney U test.

After completion of imaging, mice were sacrificed and the brains were collected. Brain tissue inflammation was measured by ionized calcium-binding adaptor molecule 1 (Iba1) immunofluorescence (percentage of area covered by positive staining). As expected, first-line TB treatment (isoniazid + rifampin + pyrazinamide) significantly reduced tissue inflammation compared to untreated animals (*P* < 0.001), which was further reduced in the dexamethasone arm (*P* < 0.001) (Fig. 4). Importantly, aspirin, bestatin, imatinib, roflumilast, palacaparib, pomalidomide, or semaglutide (*P* < 0.002) arms demonstrated further significant reductions in tissue inflammation, compared to the first-line TB treatment alone arm. Similar trends in brain tissue inflammation were observed in mice receiving TB treatment (isoniazid) with or without HDTs (**Fig. S12**). We investigated serum glial fibrillary acidic protein (GFAP) in CSF as a clinically relevant biomarker of brain injury^39,40^, which followed the trend noted above (**Fig. S13**).

#### Ex vivo immune-vascularized 3D human brain organoid model of TB meningitis

To evaluate the effect of the seven promising HDT classes on human brain resident immune cells, in particular microglia, we employed a strategic investigation using *M. tuberculosis*-infected human microglia in an *ex vivo* immune-vascularized 3D human organoid model. The brain organoid was generated by differentiating human induced pluripotent stem cells (iPSCs) into multipotent forebrain neural stem and neural progenitor cells. In parallel, yolk sac-like tissues from iPSCs were used to generate mesodermal derivatives resembling early extraembryonic hemato-vascular lineages, including CD31 + endothelial cells and CD45 + hematopoietic progenitors, which further differentiated into microglia and pericyte-like cells. Both the mouse brain and human brain organoid models used in this study exhibit comparable cellular complexity, encompassing key brain-resident cell types relevant to TB meningitis pathophysiology, including microglia, endothelial cells, and pericytes which are critical for modeling infection related neuroinflammatory responses (**Fig. S14**).

Preliminary viability assays were performed using *M. tuberculosis*-infected human microglia to establish the ideal multiplicity of infection (MOI) and time-points after infection (**Fig. S15**). Co-incubation of each individual HDTs with TB treatment (isoniazid) at the physiologically relevant, expected human brain tissue concentrations achieved with clinically relevant dosing, was performed (**Table S2, S3**). TB treatment alone reduced the Iba1 expression compared to untreated controls, which was further reduced with the addition of the promising HDTs tested compared to TB treatment alone in a dose-dependent manner (**Fig. S16**). Based on these data, we chose the physiologically relevant concentrations (**Table S3**). Using mean fluorescence intensity (MFI) of Iba1 all promising HDT classes significantly reduced microglial activation (*P* < 0.030) (**Fig. S17**). On the contrary, montelukast increased microglial activation, and overall, these results were consistent with the mouse studies.

**Figure 5:**
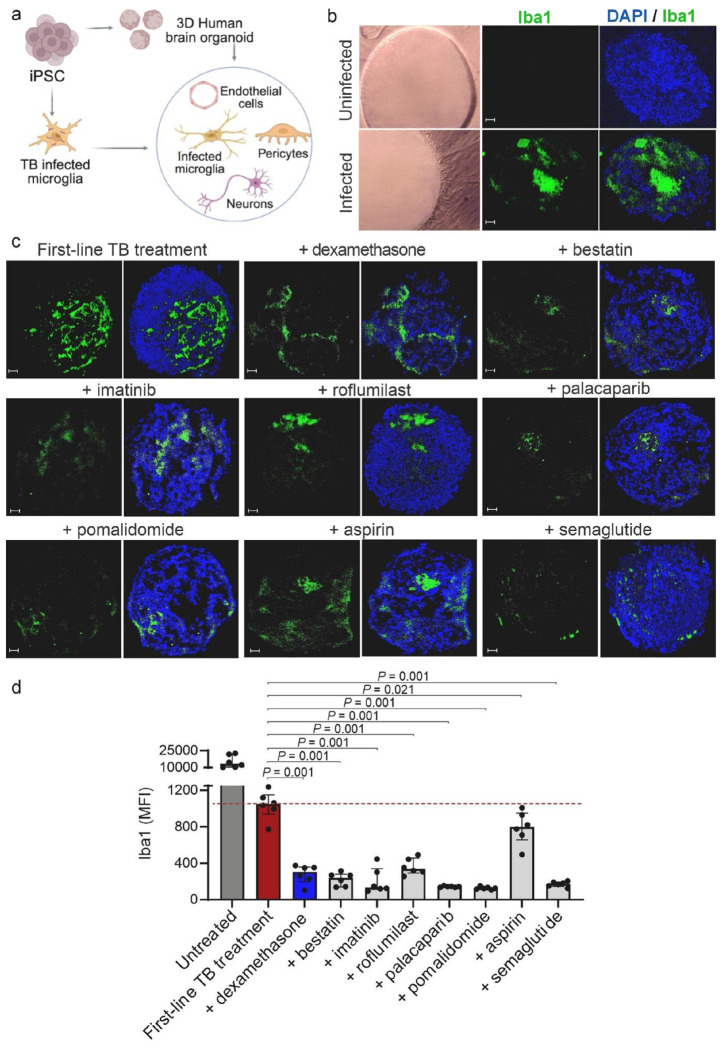
Human brain organoid model of TB meningitis. (**a**) Human immune-vascularized 3D brain organoids were developed by differentiating human induced pluripotent stem cells (iPSCs) into neural cells and mesodermal derivatives, including endothelial cells and CD45^+^ hematopoietic progenitors. The mesodermal derivatives were further differentiated into microglia and pericytes. Microglia were infected with *M. tuberculosis* and incorporated into the assembled organoids. (**b**) Optical microscopy showing the brain organoid before (top left) and after (bottom left) the incorporation of *M. tuberculosis*-infected microglia. Activated microglia (Iba1, green and DAPI for nuclei, blue) are noted in the organoid by confocal microscopy (middle and right panels) (5x magnification, scale bar = 50 μm). (**c**) Representative Iba1 immunofluorescence and merged DAPI + Iba1 images from *M. tuberculosis*-infected brain organoids after treatment with first-line TB treatment (isoniazid + rifampin + pyrazinamide), with or without adjunctive HDTs (5x magnification, scale bar = 50 μm). (**d**) Quantification of Iba1 mean fluorescence intensity (MFI) in organoids. The dashed line represents the median value for the first-line TB treatment group. Each dot represents a single brain organoid section, with at least 3 organoids per group. Data are presented as median and IQR. Statistical comparisons were made using the Mann-Whitney U test. Panel a was created in BioRender. Masias, Y. (2026) https://BioRender.com/jpy801h

Next, we performed studies with the brain organoid model where *M. tuberculosis*-infected microglia were incorporated into the assembled human brain organoids to generate a complex model comprising immune and non-immune cells and probed with multi-channel immunofluorescence (**Fig. S18**). *M. tuberculosis*-infected, but untreated organoids induced strong microglial activation, marked by increased Iba1, CD11b, and TMEM119 expression (**Fig. S19**). While the first-line TB treatment (isoniazid + rifampin + pyrazinamide) alone reduced Iba1 levels, the addition of the promising HDTs at physiologically-relevant concentrations (**Table S3**) produced a more pronounced suppression of microglial activation (Fig. 5, S20). Quantitative analysis confirmed significant reductions in Iba1 MFI with each HDTs compared with first-line TB treatment alone (*P* < 0.021) (Fig. 5). Similar trends were observed in brain organoids receiving TB treatment (isoniazid) with or without HDTs (**Fig. S21, S22**).

### Peripheral blood mononuclear cells from patients with TB meningitis

Finally, we examined the effects of the seven promising HDT classes on peripheral immune cells, specifically PBMCs, which infiltrate the CNS and play a critical role in the pathogenesis of TB meningitis^22–24^. PBMCs were collected from eight patients with confirmed TB meningitis and two healthy controls. The median age was 39 years (IQR, 33–41) and 60% (n = 6) were female (**Table S4**). Half of patients with TB meningitis were PLWH. All of them were on antiretroviral therapy and exhibited Immune Reconstitution Inflammatory Syndrome (IRIS). The other half with TB meningitis exhibited paradoxical reactions. Importantly, samples used in this study were collected during a follow-up visit, and none of the patients were receiving corticosteroids at that timepoint.

The PBMCs were cultured and re-exposed to live *M. tuberculosis* to induce the expression of a specific mycobacterial immune response (**Fig. S23**). After washing off the bacteria, the cells were treated with first-line TB treatment (isoniazid + rifampin + pyrazinamide), with or without HDTs at physiological and clinically relevant doses (**Table S3**) and analyzed using multiparametric flowcytometry (**Fig. S24**). Most of the cells recovered were CD4 + T-cells (median 32.7%, IQR 32.2–33.5%) and CD8 + T-cells (median 27.9%, IQR 26.6–29.5%), with no significant differences in the proportion of cells induced by HDT treatments (**Fig. S25**).

**Figure 6:**
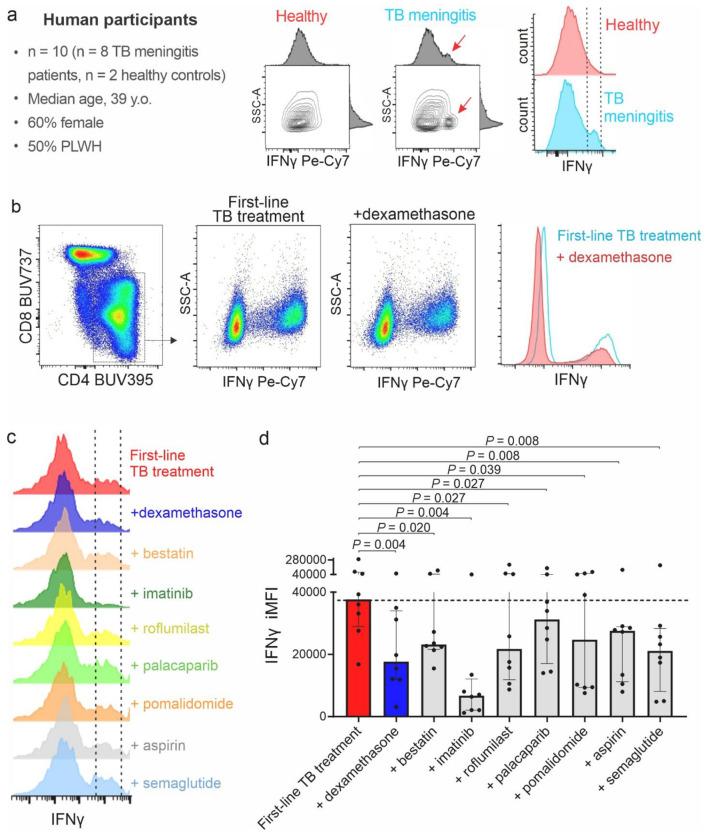
Effect of HDTs on PBMCs during TB meningitis. The effect of HDTs on immune cells that would potentially infiltrate the central nervous system during TB meningitis was evaluated using PBMCs from patients with TB meningitis (n = 8 patients and n = 2 healthy controls). PBMCs were isolated through a density gradient protocol, stimulated through exposure to live *M. tuberculosis*. (**a**) Differences between IFNγ responses of CD4+ T-cells from patients with TB meningitis and healthy controls is shown as contour and histogram plots. PBMCs were co-incubated with first-line TB treatment (isoniazid + rifampin + pyrazinamide) with or without HDTs. (**b**) Differences between IFNγ responses of CD4+ T-cells treated with first-line TB treatment with and without adjunctive dexamethasone is shown as density and histogram plots. (**c**) Histograms illustrating the effect of adding each HDTs to the first-line TB treatment on IFNγ responses of CD4+ T-cells from patients with TB meningitis. (**d**) Quantification of the IFNγ response as integrated Mean Fluorescence Intensity (iMFI) of CD4+ T-cells from patients with TB meningitis (n = 8 participants). The dashed line represents the median value for the first-line TB treatment group. Each dot represents a single patient. Data are represented as median and IQR. Statistical comparisons were made using the Wilcoxon test. PLWH, patients living with HIV.

Flow cytometry analyses showed the induction of proinflammatory cytokines, specifically a strong IFNγ expression on CD4 + T-cells upon re-exposure to live *M. tuberculosis* on PBMCs from patients with TB meningitis (Fig. 6a). Adjunctive HDTs decreased both the expression and the proportion of IFNγ positive CD4 + T-cells, evaluated by the IFNγ integrated MFI (iMFI) (Fig. 6b–d). Other proinflammatory cytokines, including IL-1β and TNFα, followed a similar trend, with decreased expression in response to adjunctive HDTs. In contrast, IL-2 and IL-10 showed minimal changes (**Fig. S26, S27**).

A similar approach was applied using PBMCs isolated from the mouse model of TB meningitis (n = 40 animals) (**Fig. S28-S30**). Consistent with the results from human PBMCs and aligned with the brain organoid studies, adjunctive HDTs were associated with downregulation of the proinflammatory response, such as IFNγ in CD4 + T-cells (**Fig. S31**), as well as other cytokines such as IL-1β, TNFα, and IL-4 (**Fig. S32, S33**).

## DISCUSSION

The treatment of TB meningitis remains challenging due to the inconsistent and suboptimal penetration of many TB antibiotics into the brain^7,8^, and current efforts to optimize antibiotic regimens have thus far only demonstrated partial success in clinical trials. For example, while higher doses of rifampin are being evaluated to overcome its limited CNS penetration; clinical trial data regarding efficacy remain conflicting^12,13,16,41,42^. While increasing rifampin dosing was shown to improve mortality or neurological outcomes in phase II clinical trials in adults and children respectively^12,13^, these benefits were not translated into improved clinical outcomes (mortality or neurological deficits) in a recent larger clinical trial^16^. Although the exact reason(s) for this lack of benefit from high-dose rifampin remain unknown, reduced corticosteroids exposure resulting from rifampin-induced hepatic clearance, has been postulated as a leading factor. Similarly, clinical studies have shown that the LTA4H genotype (leukotriene pathway) modulates intracerebral inflammation with maximal benefit of corticosteroids noted in TB meningitis patients with the hyperinflammatory TT genotype^14,15^. However, these benefits were again not translated into improved clinical outcomes (mortality or neurological deficits) in genotype-stratified TB meningitis patients expected to benefit differentially from adjunctive corticosteroids in a recent, larger clinical trial^17^. Together, these findings underscore a critical unmet therapeutic need for developing new HDTs that can more selectively modulate neuroinflammation without broadly suppressing protective immune responses. With this understanding, HDTs have emerged as an essential rather than optional strategy to develop new treatments for TB meningitis. Accordingly, we investigated a panel of 12 immunomodulatory drugs, representing 11 different immunomodulatory drug classes, which are either FDA-approved or in advanced clinical development for other indications, as potential adjunctive treatments to replace corticosteroids for the management of TB meningitis.

Extensive studies using clinical endpoints, with longitudinal monitoring, were performed in a mouse model of TB meningitis. The mouse studies were complemented by parallel mechanistic studies to evaluate the effects of these HDTs (at expected human tissue concentrations) on both human brain resident and peripheral immune cells, in a newly developed 3D immune-vascularized brain organoid model of TB meningitis and PBMCs from patients with TB meningitis, respectively. The murine model enabled the evaluation of mortality, neurological deficits and MRI-based brain imaging that mirror key clinical trial endpoints, while also correlating these findings with post-mortem immunopathology assays, which are not feasible in humans. Human equipotent drug dosing was utilized to ensure that these findings were relevant to the clinically approved (or proposed) dosing for these immunomodulatory drugs. Human organoids comprising endogenous human neural, vascular, and immune cells, enabled direct assessment of inflammatory responses within the human brain. Because the pathogenesis of active TB meningitis involves both CNS-resident and peripheral immune mechanisms^22–24^, the complementary use of brain organoids and PBMCs allowed us to capture these interacting compartments and demonstrate human-relevant immunomodulatory effects. Notably, these complex 3D systems provide a human-based system to interrogate immune-vascular mechanisms underlying neuroinflammation^43,44^, as a non-animal-based Novel Alternative Methods or New Approach Methodologies (NAMs) with extensive human-relevant applications^45^. This cross-species multi-model analysis strengthens the translational relevance of our findings by bridging mechanistic insight with clinical applicability.

To rationally prioritize candidates, we performed initial studies in an established mouse model that recapitulates the histopathological and imaging features of TB meningitis^25–27^. We evaluated each HDT as an adjunct to isoniazid alone, using relevant drug dosing informed by existing *in vitro*, preclinical, and clinical pharmacokinetic data, including evidence of brain penetration. This approach enabled meaningful rank-ordering of HDTs based on clinically-comparable outcomes, which would not be feasible with studies using the first-line TB treatment (isoniazid + rifampin + pyrazinamide), which is highly efficacious and would require an unmanageable large sample size to detect any additional therapeutic benefit^30,31^. Our approach also focused on modulating host responses during the initial phase (2-weeks) of TB treatments, as this period is associated with high mortality in patients with TB meningitis, and thus an ideal time for intervention^12,22,28,29^. The investigators were blinded to the treatment assignments for these studies. Notably, we identified several HDTs including bestatin (protease inhibitor), imatinib (tyrosine kinase inhibitor), roflumilast (PDE-4 inhibitor), or palacaparib (PARP-1 inhibitor) that reduced both mortality and neurological deficits, when compared with dexamethasone. In addition, a broader set of HDTs, thalidomide / pomalidomide (IMiDs), and semaglutide (GLP-1 receptor agonist) markedly reduced severe neurological deficits compared with our control (isoniazid); importantly, these neurological benefits were not observed with adjunctive dexamethasone, consistent with clinical data and highlighting therapeutic effects beyond the current standard of care^11,17^. Further validation of the therapeutic efficacy was performed in animals receiving adjunctive HDTs with the first-line TB treatment. Several HDTs preserved the immunomodulatory benefits observed when used in combination with isoniazid alone, indicating that their effects are not masked by potent antimicrobial treatment. Palacaparib, bestatin, roflumilast, and pomalidomide consistently reduced brain MRI contrast enhancement and microglial activation compared with the first-line TB treatment alone, while semaglutide and imatinib showed more modest effects. The modest additive effects of imatinib when used with the first-line TB treatment versus isoniazid alone could be explained due to the increased metabolism of imatinib when co-administered with rifampin^46^. Adjunctive use of other tyrosine kinase^47^ and PDE-4 inhibitors^31^ have shown to reduce pulmonary inflammation in animal models of TB, and improve lung function in patients with pulmonary TB^48^. Interestingly, pyrazinamide, an important TB drug, may also influence its effects through PARP-1 inhibition^49^. However, addition of palacaparib (PARP-1 inhibitor) to the first-line TB treatment (which includes pyrazinamide), substantially and further reduced neuroinflammation in our mouse, human brain organoid and human PBMC studies. Although, initial studies with thalidomide at a high-dose (24 mg/kg/day) demonstrated worse outcomes in children^50^, recent observational studies have shown that thalidomide at a dose of 3–5 mg/kg/day is safe and improves outcomes in children with CNS TB-related complications^20^. Thalidomide is readily available and in our mouse studies, adjunctive thalidomide at a human equipotent dosing of 3–5 mg/kg/day, was most effective in reducing neurological deficits, with mortality rates similar to dexamethasone. While adjunctive high-dose aspirin (500–1000 mg) is being used to target inflammation in TB meningitis clinical trials^51^, and we modeled using high-dose aspirin in our studies reported here, clinical evidence has been mixed, with no consistent benefit demonstrated in either children (SURE trial) or adults^52,53^. Similarly, although retrospective cohort studies and case series have demonstrated benefit of infliximab and montelukast as rescue therapies for paradoxical reactions or IRIS^19,54,55^, our findings show that infliximab did not reduce mortality nor improve neurological outcomes, while montelukast was the poorest-performing HDT and in some measures worsened neuroinflammatory outcomes. These findings highlight the selective pathways that may be critical for therapeutic benefit in TB meningitis, and that upfront HDT treatments may be different from treatments in later inflammatory reactions, which may not fully reflect early CNS-resident immune responses.

Building on this approach, we employed human microglial cultures to test these HDTs at physiological concentrations which revealed that, except montelukast, all the tested HDTs reduced microglial activation compared with isoniazid alone, enabling selection of the most physiologically relevant dose for downstream studies. Similarly, when tested at chosen clinically achievable concentrations, all the HDTs, except montelukast, significantly suppressed Iba1 expression in *M. tuberculosis*-infected human microglia. Extending these observations to a more human-relevant context, we next incorporated these *M. tuberculosis*-infected human microglia into the human brain organoid model, enabling evaluation of HDT effects within a multicellular neural environment in combination with either isoniazid alone or first-line TB treatment. Across both treatment settings, most HDTs significantly reduced microglial activation within infected organoids, as reflected by decreased Iba1 signal, whereas montelukast again failed to confer considerable benefit. Notably, the preserved efficacy of these HDTs in organoids treated with the first-line TB treatment demonstrates that their immunomodulatory effects are not masked by potent antimicrobial activity or within complex, human brain-like tissue architectures.

Finally, we used PBMCs derived from both patients with TB meningitis and the mouse model of TB meningitis to demonstrate the anti-inflammatory effects of these HDTs on the immune response to *M. tuberculosis*. Because corticosteroids are recommended and administered early to patients with confirmed TB meningitis, we used samples from patients in cohorts with paradoxical reactions and IRIS, obtained at a follow-up timepoint after corticosteroids were discontinued. Of note, all PLWH in this cohort were on anti-retroviral treatments for several weeks at this follow-up timepoint. These cells were re-stimulated with live *M. tuberculosis* and exhibited a strong IFNγ response. This system demonstrated that adjunctive HDTs at physiological doses modulated the inflammatory response in peripheral immune cells, which play a critical role both in the peripheral compartment and once they infiltrate the CNS^22–24^. Importantly, these findings were consistent, though not identical, between human and mouse cells, demonstrating a conserved anti-inflammatory effect despite the heterogeneities across species and highlighting the clinical relevance of these findings.

Our study has some limitations. Pharmacokinetic and metabolic differences between mice and humans - particularly for drugs affected by rifampin-mediated cytochrome P450 induction that may require dosing optimization or rifampin-sparing regimens^27,46,56–58^. In addition, human immune-vascularized brain organoids do not capture full patient-level genetic heterogeneity, and PBMCs obtained at follow-up from a cohort exhibiting paradoxical reactions and/or IRIS may not fully reflect early CNS-resident immune responses. However, our human brain organoid studies did capture the early CNS-resident immune responses, and the effects of the HDTs were consistent with the results noted from patient- as well as mouse-derived PBMCs. Of note, mouse-derived PBMCs also reflected early immune responses. Also, the small sample size further limits the ability to perform multivariable analysis, underscoring the importance of integrating these data with animal models. Future studies expanding patient-derived brain organoids and PBMC analyses to capture genetic, immunological, and disease-stage heterogeneity, particularly during acute disease, would further improve the translational relevance and guide precision clinical trials.

In conclusion, this study is one of the most comprehensive evaluations of promising HDTs across complementary experimental systems - including mouse models with clinically-relevant endpoints (mortality, neurological deficit, brain MRI), human immune-vascularized brain organoids, and patient-derived PBMCs. These approaches demonstrate that several targeted immunomodulatory drug classes outperform dexamethasone in reducing neuroinflammation, mortality risk, and neurological injury in TB meningitis. By enabling mechanism-specific modulation of pathogenic inflammation without compromising antimicrobial efficacy, these findings provide a strong translational rationale for advancing select immunomodulatory drug classes into early-intervention clinical trials to improve survival and long-term neurological outcomes.

## METHODS

### Animal studies

All protocols were approved by the Johns Hopkins University Biosafety, Radiation Safety, Animal Care and Use Committees (MO19M382).

Female C3HeB/FeJ mice (6–8 weeks old, Jackson Laboratory) were infected (titrated frozen stocks with ~ 6.5 log_10_ CFU of *M. tuberculosis* H37Rv) via a burr hole (Micro-Drill Kit, Braintree Scientific Inc.) using a Hamilton syringe (Hamilton, 255 88000) and stereotaxic instrument (David KOPF Instruments, model 900, coordinates 0.6 mm dorsal to bregma, 1.2 mm lateral to middle line, and 2 mm ventral). Sham mice that received intracranial injections of sterile PBS, rather than bacteria, served as controls to distinguish the effects of the infection from those due to the intracranial injection procedure itself. All animals were housed in controlled light and temperature rooms without cross-ventilation in a BSL-3 facility with unrestricted access to both food and water.

#### Treatments:

All drugs were purchased from MedChem and were either dissolved or suspended in water per the manufacturer instructions. All drugs and antibiotics were prepared and administered daily, five days per week, except for semaglutide and infliximab, which were administered every other day and once weekly, respectively, at the indicated dose and route. The immunomodulatory drug doses were chosen to simulate clinically relevant doses for immunomodulatory effects in humans, while accounting for mouse pharmacokinetics (**Table S1, S2**). The 5-day-on / 2-day-off schedule is standard for treatments in the mouse models of TB to approximate continuous daily therapy while accounting for animal handling and welfare. In addition, clinical guidelines and extensive programmatic experience interpret directly observed therapy given 5 days per week as equivalent to 7-day “daily” therapy, with comparable treatment success when the total number of doses is adjusted accordingly. TB antibiotics were administered at the following doses: isoniazid (10 mg/kg/day), rifampin (10 mg/kg/day), and pyrazinamide (150 mg/kg/day). Bacterial burden was quantified in whole organs (brain, lung and spleen) as CFU following two weeks of treatments using 7H11 plates supplemented with activated charcoal. Plates were incubated at 37°C for three weeks before CFU were counted.

##### Survival and neurological signs assessment

Cohorts of animals were individually marked for identification and randomly assigned to each treatment group. Mice were evaluated each week for survival, weight changes and development of neurological signs. Evident head tilt and partial or complete unilateral or bilateral limb paralysis were considered as development of severe neurological signs. The investigators were blinded to the treatment assignments for the survival and neurological deficit studies.

##### Dynamic gadolinium-enhanced brain MRI

Multislice Fast Spin Echo T1-weighted (T1w-FSE; repetition time (TR) = 1000 ms; echo time (TE) = 11 ms; number of averages = 1; field of view (FOV) = 2 × 2 cm; matrix size = 256 × 248; slice thickness = 1 mm; number of slices = 14–16; fat saturation applied) and FLASH Dynamic Contrast-Enhanced Magnetic Resonance Imaging (FLASH DCE-MRI; TR = 100 ms; TE = 4 ms; flip angle = 25°; number of averages = 1; FOV = 2 × 2 cm; matrix size = 192 × 100; slice thickness = 1 mm; number of slices = 11; time of scan = 5 or 20 minutes, intervals of 10 seconds) were performed before and after intravenous injection of gadolinium-based contrast (Magnevist, Bayer; 0.2 mmol/kg) to assess brain changes in response to treatment. Imaging was performed using a 7T MRI system (MRS DRYMAG, MRSolutions, UK) equipped with a Quadrature 20 mm-diameter mouse head coil and custom-built animal biosafety level-3 (ABSL-3) MRI-compatible biocontainers (**Fig. S8**). VivoQuant, Preclinical ScanTM and PowerscanTM were used for MRI data analysis. AUC were calculated for the segmented whole-brain region from dynamic MR images by summing the difference maps (post-/pre-contrast) across all experimental timepoints during the 5–20 min acquisition and normalizing by background. Delta maps were generated by digitally subtracting the pre-contrast image from the post-contrast image after manual segmentation of the whole brain.

###### Histopathology and immunofluorescence:

Mice were sacrificed two weeks after treatment initiation and organs (brain, lung and spleen) were harvested and fixed after systemic perfusion with 4% paraformaldehyde. Multiple random slides were obtained from each tissue sample, stained and digitally scanned. Slides were incubated overnight at 4°C with a primary antibody against Iba1 (Thermo Fisher MA5-36257, 1:500). After washing the sections, a secondary goat Alexa-fluor 488 antibody (Thermo Fisher, A11034, 1:100) was used to incubate the tissues for 2 h at room temperature. Sections were washed and mounted with 4’,6-diamidino-2-phenylindole (DAPI, Thermo Fisher, ProLong^™^ Gold Antifade Mountant with DNA Stain DAPI). Leica DM6B system (Leica) confocal microscope was used at 40x resolution to image. For data analysis, ten to 15 images per sample were processed in FIJI ImageJ to determine the % area staining for Iba1.

##### CSF and brain homogenate assays

Animals were sacrificed and CSF, and brain tissue were extracted. Western blot analysis was performed on brain lysate using a standardized protocol using specific primary and secondary antibodies (**Table S5**). The protein bands were visualized on the membranes using chemiluminescent substrates (Supersignal West Pico maximum sensitivity substrate, cat. no. 34580) and analyzed using FIJI ImageJ (NIH).

### Human microglia

Preliminary studies were performed to determine the optimum MOI and timepoint for downstream assays (**Fig. S15**). Cell viability was assessed using lactate dehydrogenase release assay (CyQUANT LDH Cytotoxicity Assays, cat. no. C20301). Based on the results, MOI 5 at 24 hours was selected for all subsequent studies.

### Human brain organoid model of TB meningitis

Human iPSCs were maintained in DMEM/F12 supplemented with KnockOut Serum Replacement, FGF2, GlutaMAX, non-essential amino acids, and 2-mercaptoethanol under Johns Hopkins ISCRO approval. Neural differentiation was initiated by collagenase-mediated detachment and suspension culture, followed by dual-SMAD inhibition (Noggin, dorsomorphin, SB431542). Embryoid bodies were plated on Matrigel or laminin to generate neural rosettes, from which rosette-derived neural aggregates (RONAs) were isolated and maintained as neurospheres in Neurobasal medium. Neural rosettes were expanded into glial progenitor cells and differentiated into astrocytes using defined induction and maturation media, including CNTF, over a 4-week period. Human iPSCs were differentiated into yolk sac-like tissues using staged cytokine-driven protocols to generate macrophage-like microglial progenitors, along with endothelial and pericyte populations. Floating microglial progenitors were collected after 25 days and maintained in microglia growth medium for downstream studies^59^. *M. tuberculosis*-infected microglia were incorporated into the organoids containing endothelial cells and pericytes. Whole organoids were treated at the specific drug concentrations for 24 hours and then formalin fixed and stained for multiple markers (**Table S5**).

### PBMCs obtained from patients with TB meningitis

Human studies were approved by the NIH Institutional Review Board, and studies with de-identified samples were performed. PBMCs were collected from eight patients with confirmed TB meningitis and two healthy controls from prospective observational studies (NCT00286767, NCT02147405, NCT04052022) at the National Institutes of Health (NIH). All participants provided written informed consent. The samples were collected during a follow-up visit, and none of the patients were receiving corticosteroids at that time (**Table S4**). One day prior to infection, PBMCs were thawed, washed and cultured in complete RPMI (cRPMI) supplemented with 10% FBS, 1 mM sodium pyruvate, and 1% non-essential amino acids at 37°C in 5% CO_2_ (**Fig. S23**). *M. tuberculosis* was cultured for two weeks in Middlebrook 7H9 medium. Cells suspended in the supernatant were separated and adherent cells (mainly monocytes) were infected for 4 hours at a multiplicity of infection (MOI) of 1:5 based on 10% monocyte number. After infection, bacteria were washed off, and the cells in the supernatant were returned to the culture and resuspended in cRPMI supplemented with the drugs at the indicated concentrations (**Table S3**). After 20 hours of treatment, cells were stimulated with Cell Stimulation Cocktail (e.g., PMA/Ionomycin) together with GolgiStop/GolgiPlug (BD Biosciences) for 4 hours to block cytokine secretion.

#### Mouse PBMCs:

Peripheral blood was collected from mice two weeks after TB meningitis infection. PBMCs were isolated using HISTOPAQUE^®^-1077 and stored at −80°C. One day prior to infection, PBMCs were thawed and cultured in cRPMI at 37°C in 5% CO_2_ (**Fig. S28**). PBMCs were infected for 4 hours at a multiplicity of infection (MOI) of 1:5 based on 10% monocyte number. After infection, cells were washed and resuspended in cRPMI with drugs at the indicated concentrations (**Table S3**). After 24 hours of treatment, supernatants were collected for cytokine quantification. Cells were then resuspended in medium and stimulated with Cell Stimulation Cocktail (e.g., PMA/Ionomycin) together with GolgiStop/GolgiPlug (BD Biosciences) for 4 hours to block cytokine secretion.

##### Flow Cytometry

Antibodies used for human and mouse staining are listed in **Table S6**. Cells were washed in PBS and stained for viability using Fixable Viability Dye Live/Dead Blue (Invitrogen, L23105). After washing with FACS buffer (PBS + 0.5% BSA), Fc blocking was performed for 10 minutes at room temperature using Human Fc Block (BD Biosciences, 564220) or Mouse Fc Block (BD Biosciences, 553142). Surface staining was performed in 100 μL of FACS buffer using a surface antibody cocktail for 20 minutes at 4°C. Cells were then fixed and permeabilized using the Fixation/Permeabilization Solution Kit (eBioscience, 00-5521-00). Following fixation, cells were washed with Permeabilization/Wash buffer (eBioscience, 00-8333-56) and Cytflox/Cytoperm (BD Biosciences, 554714); intracellular staining was performed in 100 μL of the same buffer with an intracellular antibody cocktail for 1 hour at 4°C. After staining, cells were resuspended in FACS buffer and acquired on a 5-laser Cytek Aurora spectral flow cytometer (Bayview Immunomics Core, Johns Hopkins Bayview Campus, MD). Data were analyzed using FlowJo v11 (BD) with gating strategies shown (**Fig. S24, S29**). High-dimensional analysis of cell phenotypes was performed using the FlowJo plugins DownSample v3 and UMAP. Integrated mean fluorescence intensity (iMFI) was calculated by multiplying the mean fluorescence intensity of each marker by the percentage of positive cells.

### Statistical analysis

Data was analyzed using Prism 10 Version 10.1.1 (GraphPad). Bacterial burden (CFUs) are represented on a logarithmic scale (base 10) as mean ± SD and all other data are represented as median ± IQR. Comparisons were made using a single-tailed Wilcoxon, Mann-Whitney U or Chi-square test to the predefined control group. RR and 95% CI were calculated using the Katz log rank test. *P* values ≤ 0.05 were considered statistically significant. No sex-based analyses were performed because the study design was not explicitly set up to examine sex differences.

## Supplementary Material

This is a list of supplementary files associated with this preprint. Click to download.

• 20260227Supplementarydata.docx

## Data Availability

All data are available in the main text or the supplementary materials. Source data are provided with this paper.
